# Torsion d'annexe après hystérectomie abdominale: une première observation

**DOI:** 10.11604/pamj.2015.22.9.7759

**Published:** 2015-09-07

**Authors:** Abderrahman Elhjouji, Othman Zahdi, Hicham Baba, Said Belhamidi, Ahmed Bounaim, Abdelmounaim Aitali, Khalid Sair

**Affiliations:** 1 Service de Chirurgie Viscérale I, Hôpital Militaire d'Instruction Mohamed V, Rabat, Maroc

**Keywords:** Annexe, torsion, hystérectomie, adnexa, torsion, hysterectomy

## Abstract

La torsion d'annexes survient classiquement sur ovaires kystiques ou tumoraux. De rares cas de torsion ont été rapports dans la littérature après hystérectomie laparoscopique. Nous rapportons la première observation de torsion d'annexe survenant sur annexe normale après hystérectomie abdominale et décrivons les particularités de cette forme clinique.

## Introduction

Véritable urgence chirurgicale, la torsion d'annexes sur un ovaire normal est une entité peu fréquente voire même exceptionnelle. Le diagnostic préopératoire est difficile du fait de signes non spécifiques. A notre connaissance, de rare cas de torsion d'annexes ont été rapportés dans la littérature après hystérectomie laparoscopique, aucun cas après hystérectomie par laparotomie. Nous rapportons une première observation de torsion d'annexe survenant sur ovaire normale après hystérectomie par laparotomie et décrivons les particularités de cette forme clinique.

## Patient et observation

**Figure 1:**
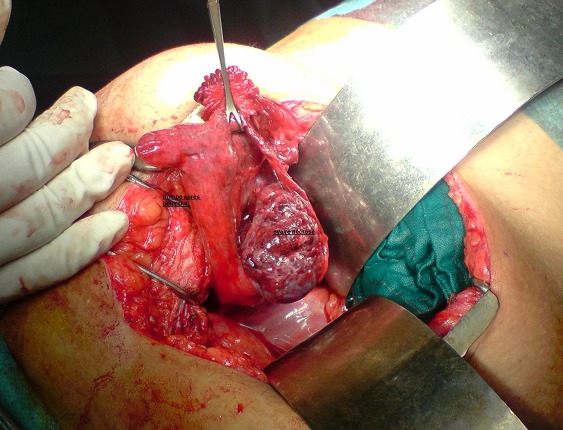
Image opératoire après détorsion de l'annexe montrant la nécrose de l'ovaire droit

Une patiente de 62 ans, ménopausée, ayant eu 4 ans auparavant une hystérectomie par voie abdominale pour utérus polymyomateux. Elle s'est présentée aux urgences pour douleurs abdominales hypogastriques remontant à 3 jours avec vomissement et fièvre sans troubles du transit. A l'admission, la patiente était fébrile à 38.7°C. L'examen abdominal a retrouvé un abdomen sensible légèrement ballonné sans défense. La biologie a confirmé l'existence d'un syndrome infectieux, avec CRP et globules blancs élevés. Une échographie réalisée n'a pas révélé d'anomalies en dehors d'un épanchement abdominal de faible abondance. Le scanner abdomino-pelvien a confirmé le même constat sans orienter vers la cause. Nous avons décidé de réaliser une c'lioscopie diagnostique. A l'exploration, nous avons trouvé un épanchement séro-hématique avec une agglutination des anses au niveau de la fosse iliaque droite. Après conversion à cause des adhérences post-opératoires rendant la dissection difficile et dangereuse, une torsion d'annexe à double tour de spire a été retrouvée, avec une nécrose de l'ovaire droit (Figure 1). On a décidé de réaliser une annextectomie droite. Les suites opératoires ont été simples et la patientes est sortie de l'hôpital à J + 2. L'examen anatomopathologique de la pièce n'a pas trouvé de lésion au niveau de l'ovaire mise à part les signes de nécrose.

## Discussion

A notre connaissance, celle-ci est la première observation rapportée de torsion d'annexe après hystérectomie abdominale. De rares cas de torsion après hystérectomie coelioscopique ont été rapportés [1]. Il s'agit d'ailleurs de la seule série retrouvée dans la littérature. Dans cette série, l’âge moyen des patientes était de 45.92 ans. Notre patiente était âgée de 62 ans. La torsion est survenue 4 ans après l'hystérectomie et a touché l'annexe droite. Dans la série de Mashiach et al. [1], le délai était de 2.64 ans et le coté gauche était plus souvent atteint. Le risque de torsion d'annexe après hystérectomie coelioscopique serait probablement du à la formation de moins d'adhérences par rapport à la voie ouverte. Ceci est appuyé par plusieurs éléments. La précision des gestes utilisant moins de sutures et de pincement des tissus, la dissection douce et l'utilisation de la coagulation bipolaire causent moins de traumatismes péritonéaux [2, 3], moins d'infections et d'hémorragie [4, 5], et moins d'iléus postopératoire [6]. En cœlioscopie, le contenu intra-abdominal surtout le péritoine a moins de contact avec les corps étrangers (champs, compresses..) qui favorise la formation d'adhérences. En plus de la théorie d'adhérences, Mashiach et al. expliquent ce risque de torsion après hystérectomie laparoscopique par rapport à la voie abdominale par deux autres éléments [1]: durant l'hystérectomie laparoscopique, le ligament large est coagulé et disséqué et non lié ce qui rend l'ovaire restant plus mobile. De plus, l'ovaire subit moins de traumatismes donc il vie plus longtemps, continue à ovuler, demeure lourd et peut produire des kystes, ce qui favorise la torsion. Notre observation, en plus de l'antécédent d'hystérectomie, a plusieurs particularités: Si la torsion d'annexe touche plus fréquemment des femmes jeunes en période d'activité génitale et parfois même des jeunes filles impubères mettant ainsi en jeu le pronostic fonctionnel, notre patiente était âgée et ménopausée. Les examens d'imagerie n'ont pas permis le diagnostic ce qui insiste sur l'intérêt de la cœlioscopie. En effet, L'apport diagnostique considérable de la cœlioscopie a révolutionné cette pathologie et a permis de réactualiser le traitement conservateur. Aucune anomalie (kystes ou autres) favorisant la torsion n'a été retrouvée à l'examen histologique en dehors des lésions ischémiques. La fréquence des torsions après hystérectomies est probablement sous-estimée du fait de la possibilité de torsion asymptomatique ou méconnue. Cependant, contrairement à la laparoscopie où une pexie est souvent réalisée [7], celle-ci ne semble pas être justifiée après hystérectomie ouverte, par contre demeure la nécessité de réaliser un geste sur l'ovaire controlatéral (pexie ou ovariectomie) pour éviter une récidive controlatérale.

## Conclusion

Nous présentons dans cette observation pour la première fois la torsion d'annexe comme complication possible de l'hystérectomie abdominale. Une étude des dossiers d'hystérectomie abdominale et une surveillance des patientes sont nécessaires pour l’évaluation de la fréquence réelle de cette entité pathologique.
